# Immune Tolerant Chronic Hepatitis B: The Unrecognized Risks

**DOI:** 10.3390/v9050096

**Published:** 2017-04-29

**Authors:** Patrick T. F. Kennedy, Samuel Litwin, Grace E. Dolman, Antonio Bertoletti, William S. Mason

**Affiliations:** 1 Centre for Immunobiology, Blizard Institute, Barts and the London School of Medicine & Dentistry, QMUL, London E1 2AT, UK; g.dolman@qmul.ac.uk; 2 Fox Chase Cancer Center, Philadelphia, PA 19111, USA; samuel.litwin@fccc.edu (S.L.); ws_mason@fccc.edu (W.S.M.); 3 Emerging Infectious Diseases Program, Duke-NUS Graduate Medical School, Singapore; antonio@duke-nus.edu.sg

**Keywords:** hepatitis B, immune tolerant, hepatocellular carcinoma, cirrhosis, vaccine, inflammation

## Abstract

Chronic infection with hepatitis B virus (HBV) progresses through multiple phases, including immune tolerant, immune active, immune control, and, in a subset of patients who achieve immune control, reactivation. The first, the immune tolerant phase, is considered to be prolonged in duration but essentially benign in nature, lacking long-term consequences, and thus not recommended for antiviral therapy. This review challenges the notion that the immune tolerant phase is truly benign and considers the possibility that events during this phase may contribute significantly to cirrhosis, hepatocellular carcinoma (HCC), and the premature death of 25% of HBV carriers worldwide. Thus, earlier treatment than recommended by current guidelines should be considered. Low therapeutic coverage exacerbated by restrictive treatment guidelines may facilitate disease progression in many patients but also increase the risk of neonatal and horizontal transmission from untreated mothers to their children. While a prophylactic vaccine exists, there are many areas worldwide where the treatment of adults and the delivery of an effective vaccination course to newborns present difficult challenges.

## 1. Introduction

Hepatitis B virus (HBV) replicates in hepatocytes. Elevated hepatocyte turnover during HBV infection is thought to be mostly attributable, directly and indirectly, to the adaptive immune response against these infected cells. Hepatitis B is a non-cytopathic virus and thus does not directly cause hepatocyte death or marked changes in hepatocyte appearance. It is not known whether infection per se contributes to cell death over the prolonged time course of chronic infection.

Chronic HBV infection is typically acquired at birth or in early childhood, particularly in Asian and African countries where HBV is endemic. The risk of developing chronic infection after exposure drops from ~90% in neonates to 1–5% in healthy adults [1,2]. Most infections in adults are characterized by an acute symptomatic illness, which resolves within a few months with the loss of hepatitis B surface antigen (HBsAg). Failure to clear HBsAg is the hallmark of chronicity.

Chronic infections acquired perinatally or in early childhood are considered to pass through several prolonged disease phases; immune tolerant, immune active, immune control, and, in a proportion of patients, reactivation. Immune mediated liver injury is often associated with elevated serum alanine aminotransferase (ALT) levels. The immune tolerant phase is defined by high titer viremia, of ~10^9−10^ virions per mL, and normal ALT levels, suggesting an essentially healthy liver with no ostensible disease activity. Based on issues raised in this review, the idea that the liver is typically healthy (i.e. normal) in immune tolerant patients as defined by these serologic criteria is supported neither by recent nor historical data. Therefore, we believe that ‘high replicative, low inflammatory’ may be a better designation for this phase of infection [3] since it does not inherently imply that this phase of HBV infection is benign. In order to avoid any confusion throughout this review, we will retain use of the conventional designation of ‘immune tolerant (IT)’, while attempting to make clear why we believe that ‘high replicative, low inflammatory’ is more accurate for designating this phase of chronic infection.

The designation of a normal ALT has evolved over time from <50 IU/mL to <30 IU/mL in a healthy adult male and <19 in a healthy adult female [4]. Though ALT levels may also be affected by diet, alcohol consumption, fatty liver, etc., persistently elevated levels (e.g. for >3–6 months) are a warning that chronic HBV infection may have entered the immune active phase [2]. In addition, if viremia declines below 2 × 10^8^ copies/mL in IT patients, immune active hepatitis should be considered even if ALT levels are in the normal range [5]. In this regard, it should be noted that hepatocyte death via apoptosis and necroptosis has been observed during hepatitis B, with the former being prominent in mild forms of hepatitis. The quantitative contribution of these and other forms of cell death to ALT elevations in chronic hepatitis B are unknown, and it remains a concern that ALT measurements may fail to detect the mild hepatitis that has been observed histologically in IT patients [6,7] and could be contributing to disease progression during the IT phase. The most serious consequences of chronic infection are cirrhosis and hepatocellular carcinoma (HCC). Cirrhosis does not occur in the IT phase of infection, but early stages of fibrosis are often present, as we and others have reported. It remains possible that steps in the initiation, promotion, and/or progression of HCC could begin in this early phase of disease [8].

Based on data from animal models, it is thought that all hepatocytes are productively infected in the first months of some self-limiting acute and all chronic infections [9,10,11,12]. As noted above, subsequent events differ considerably. During a self-limiting acute infection, the adaptive immune response can rapidly clear the virus even after an infection of 100% of hepatocytes. The actual mechanism is controversial, as hepatocytes in the recovered liver are thought to arise from previously infected hepatocytes [13]. There is no agreement on how covalently closed circular DNA (cccDNA), the viral transcriptional template, is eliminated from these hepatocytes [14,15,16,17,18]. It is also unclear why adaptive immunity and viral clearance are delayed for weeks to months after infection of the entire hepatocyte population [10,11,12]. In any case, once initiated, the adaptive immune response can achieve viral clearance within a few weeks. Low levels of residual cccDNA may persist in the liver after resolution of self-limiting acute HBV infection [13,19,20] and are presumably responsible for (1) persistence of antiviral T cells in recovered individuals, due to the residual expression of viral proteins [21], and (2) viral reactivation during immunosuppression [22].

In contrast to a self-limiting infection, characterized by a marked and robust immune clearance phase, it is possible that chronic infection progresses directly to the IT phase, at least in many neonatal infections. During the classical immune active phase of chronic infection, adaptive immunity leads to an inflammatory response via antiviral T cells and recruitment of non-antigen specific inflammatory cells. If sustained, this inflammatory response may lead to cirrhosis. In contrast to acute infections, this immune response seldom results in viral clearance (e.g. even if virus titers drop to very low levels, the release of virus envelope particles (HBsAg) into the blood stream persists). This failure may be due in part to the ability of the liver to suppress intrahepatic immune responses [23,24]. At least early in infection, HBV chronicity may also be facilitated by hepatitis B e antigen (HBeAg). Mouse studies suggest that this secretory version of the HBV core (nucleocapsid) protein suppresses anti-HBV cellular immune responses to infections acquired neonatally from HBeAg(+) but not HBeAg(−) mothers [25,26], though whether this applies to humans and how long this effect might persist is unclear.

Ultimately, the immune active phase may progress to immune control. Biochemical flares, characterized by dramatic ALT elevations with associated and marked declines in viremia, are often observed in the transition to the immune control phase. In most cases, when the immune active phase progresses to immune control, ALT levels normalize and viremia drops below 10^4^ copies/mL [2,27]. At the same time, HBeAg usually disappears from the serum and anti-HBeAg immunoglobulins appear that are sufficient to clear residual levels of circulating HBeAg. Serum HBsAg persists. Liver disease reactivates in about a third of patients that have entered the immune control phase, with ALT elevations and an increase of virus titers above 10^4^ copies/mL. This is referred to as HBeAg(−) hepatitis [28]. Immune control and HBeAg(−) hepatitis are associated with the emergence of HBeAg(−) mutant strains of HBV, which have alterations in the core promoter and/or pre-core regions of the viral genome needed for HBeAg production. The rebound of viremia above 10^4^ copies/mL is probably due to an increase in the number of hepatocytes that are replicating HBV. Even in the absence of ALT elevations, virus titers above 10^4^ copies/mL are thought to represent ineffective immune control of the virus and to be an indicator of active liver disease [29].

**Figure 1:**
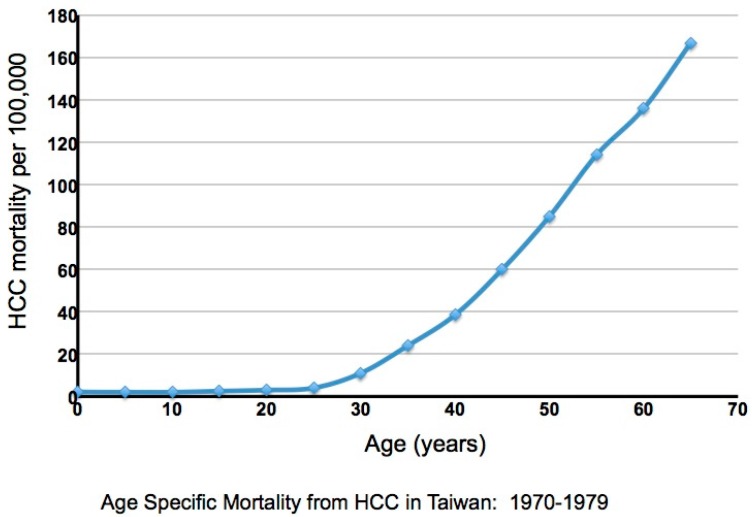
Age specific hepatocellular carcinoma (HCC) mortality in Taiwan. Adapted from Figure 2 of a paper by R.P. Beasley [31].

As noted, the complications of chronic infection are cirrhosis and HCC, with about 85% of HCCs occurring in cirrhotic livers. In Taiwan a very high incidence of HCC had been observed historically, with the incidence increasing dramatically after the age of 30 [30,31] (Figure 1). A large-scale study of 22,707 male Taiwanese government workers, in whom HBV infection is primarily perinatal, revealed that the high incidence of HCC occurs primarily in HBV carriers (i.e., HBsAg(+) individuals). Seventy cases were observed among 2027 HBsAg(+) individuals, and only one in an HBsAg(−) individual, albeit with a history of HBV infection revealed by detection of anti-HBsAg and anti-HBcAg antibodies. Overall, approximately 40% of neonatally infected male carriers will die of either HCC or cirrhosis [31]. The rate is about two- to three-fold lower in women [31]. Importantly, this study revealed a strong dependence of HCC upon age, and by inference, the duration of infection, though the timing of critical steps in oncogenesis still remains unresolved.

Another landmark study from Taiwan, REVEAL-HBV, correlated virus titers in the serum with HCC risk [27]. The study recruited older HCC-free patients of varying ages, with a minimum of 30 years, the time at which the HCC incidence undergoes a sharp rise (Figure 1). HBeAg status (+ or −) and viremia at study entry were correlated with clinical outcome after a follow-up of ~12-years. About 80% of the patients were HBeAg(−). The cumulative occurrence of HCC during follow-up was lowest (~1%) in patients with virus titers below 10^4^ copies/mL at study entry, about threefold higher in patients with virus titers between 10^4^ and 10^5^ copies/mL, about eight-fold higher in patients with titers of 10^5^ to 10^6^ copies/mL, and ~13 fold higher in patients with titers above 10^6^ copies/mL [27]. A higher risk of HCC previously reported in patients that remained HBeAg(+) [32,33] may be related to higher levels of viremia [27] and a more prolonged hepatitis in many of these patients.

## 2. Anti-Viral Therapy

Owing to the high short-term (5–10 year) risk for HCC development in patients above the age of 30 years [27,30,31], antiviral therapy to block HBV replication and reduce oncogenic progression has been studied extensively in this cohort. Therapy with pegylated-interferon alpha (peg-IFN) is typically effective only in patients in the immune active phase of infection, and, even then, conversion to the immune control phase of infection is rare, at about 30% for HBeAg(+) disease [34,35]. In this situation, pegylated interferon (peg-IFN) is thought to work by boosting the host’s immune response to HBV infected hepatocytes, leading to disease control. The response to peg-IFN in HBeAg(−) hepatitis is assessed using markers of immune control; a sustained normalization of ALT and reduction in HBV DNA (<10^4^ copies/mL). About 20% of patients maintain a sustained off-treatment response defined by these parameters [35]. HBsAg loss is considered the gold standard treatment endpoint in the management of chronic hepatitis B (CHB) and this is achieved in approximately 10% of HBeAg(−) patients three years post treatment with peg-IFN therapy [36]. When this endpoint is achieved, the risk of HCC is significantly reduced [37]. A major obstacle to the administration of peg-IFN as a first-line therapy, in addition to its limited efficacy, is that regular patient monitoring is required to identify a transition from IT (or immune control) to an immune active disease phase.

Lamivudine, the first Food and Drug Administration (FDA) approved nucleoside analogue inhibitor of HBV replication, was effective in a clinical trial of older cirrhotic patients in reducing the five year risk of HCC [38]. However, over this time interval, the emergence of lamivudine resistant virus became a major issue, as resistant virus with mutations in the viral DNA polymerase, especially the tyrosine-methionine-aspartate-aspartate (YMDD) motif, supplanted wild-type HBV (wtHBV). Viral breakthrough due to emergence of drug resistant HBV can lead to immune activation with hepatic flares, which are thought to reflect significant immune mediated liver injury.

To date, Tenofovir and Entecavir are the only nucleos(t)ide analogues for which the emergence of drug resistant strains of HBV are rare in treatment naïve patients [39,40], (~1% for Entecavir, none for Tenofovir). These drugs are considered first-line treatment options and represent the best hope of HCC prevention in a wide range of patients. For example, a recent study showed that Entecavir therapy reduced the five year risk of HCC approximately fivefold, particularly among cirrhotic patients [41], similar to historical data with lamivudine. When other factors were considered, the results with Entecavir were consistent with prediction models that considered a variety of clinical assessments, including cirrhosis, viremia, age, ALT elevations, HBeAg status, smoking, alcohol consumption, etc., as HCC risk factors (e.g. [42,43,44,45]).

Patients over 30 years old that remain persistently HBeAg(+) have an especially high life-time risk of HCC of 87%, versus 12% for patients seropositive for HBsAg but HBeAg(−) (see [32] for review). This may seem a paradox since IT patients with apparently low disease activity are HBeAg(+). The difference is believed to be due to the fact that about a third of patients progress to the immune active phase without eventually achieving HBeAg seroconversion and effective immune control of HBV replication. That is, once the immune active phase is entered, ultimate reduction of viral replication (with viremia < 10^4^ copies/mL), either by host immune control or via antiviral therapy, is essential to reduce the long-term risk of HCC.

While HBeAg(−) seroconverson is considered to end the immune active phase if virus titers stably fall below the 10^4^ copies/mL threshold, it is noteworthy that seroconversion even with this outcome might be a contributing factor in the development of HCC in some patients, especially if seroconversion is preceded by a large amount of liver turnover. For instance, during seroconversion, HBV mutants that are unable to produce HBeAg due to stop codon mutations in the signal peptide region of the HBe precursor protein or that have reduced production due to upstream mutations in the promoter for the HBe mRNA usually become the predominant form of HBV in the liver. wtHBV may also persist but at reduced levels [46]. Since all HBV susceptible hepatocytes are presumably infected and resistant to superinfection [47], the emergence of HBeAg(−) HBV probably includes immune selection against hepatocytes producing wtHBV in favor of those expressing the mutants [48]. Similar to the emergence of HBV negative hepatocytes (i.e. not supporting HBV replication), which is observed during chronic infection, immune selection for hepatocytes infected with HBeAg(−) variants of HBV could require considerable hepatocyte turnover [48] with selective emergence of initially rare hepatocyte clones due to immune escape.

## 3. When to Initiate Antiviral Therapy

Recent assessments suggest that antiviral therapy coverage is low, reaching <1% of HBV carriers (Global health sector strategy on viral hepatitis 2016–2021, World Health Organization). Thus, the vast majority of carriers experience the natural course of infection, with the inherent risk for the development of cirrhosis and HCC as first documented over 30 years ago [30,31]. The impact of this is greatest in areas of high prevalence, predominantly affecting low and middle-income countries, where access to healthcare services may be limited.

Of patients with CHB across five European countries, 45% were receiving treatment. The proportion of untreated patients that met the European Association for the Study of the Liver (EASL) criteria for initiation of treatment varied from 7% to 39% depending on country and reimbursement arrangements at the time of the study [49]. Funding restrictions for nucleos(t)ides have limited treatment access for patients with CHB in Canada [50]. In the UK, a third of patients under specialist follow-up were on treatment [51]. However, identifying the large number of individuals with undiagnosed CHB represents the greatest barrier to offering treatment in all geographic settings [52,53].

When available to patients, therapy with nucleos(t)ides will suppress virus replication at any stage of infection. However, there are still issues concerning their use that are unrelated to their effect on HBV replication. For instance, there are concerns that lifetime treatment with antivirals may have adverse, unforeseen, side effects [54]. In addition, discontinuation of therapy may lead to significant liver disease if or when virus titers rebound so that the decision to treat also implies careful monitoring post treatment cessation.

**Figure 2:**
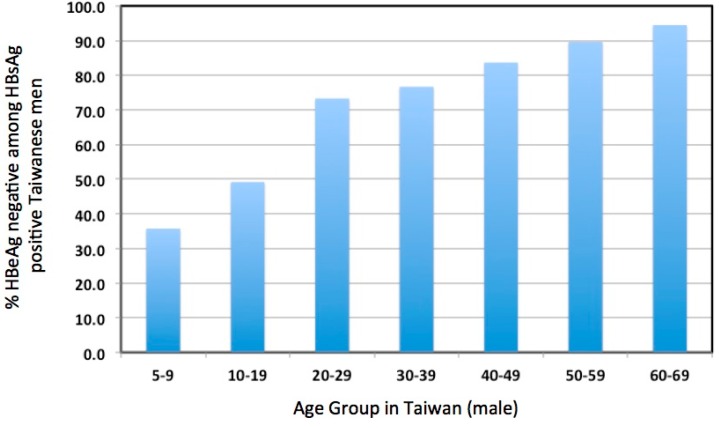
Incidence of HBeAg(−) patients among various age groups of HBsAg(+) Taiwanese males. Adapted from Table 2 of a paper by You et al. [32].

Aside from these concerns, HBV infection in the IT phase, most prevalent in young patients, has not been considered to cause sufficient liver disease to justify antiviral therapy [2,55,56,57]. For these and other reasons, patients in the IT phase of infection are usually not considered for treatment. Whether this is justified is a worrisome issue. The emphasis on older patients due to the increasing risk of HCC above the age of 30 years may reduce the amount of attention given to over 70% of young patients that enter or progress through the immune active phase to HBeAg seroconversion by age 30 (Figure 2) [32]. Our own clinical data confirms this observation; of the 366 patients between the ages of 16 and 30 years in a dedicated young adult HBV clinic, 69/366 (19%) had HBeAg (+) disease and only 32/366 (9%) could be considered to be immune tolerant based on clinical and virological parameters (i.e. virus titers above 2 × 10^8^ copies/mL and normal serum ALT levels).

Nevertheless, as discussed above, nucleos(t)ide therapy in immune active patients in their 30s or older has important benefits, including normalization of ALTs (implying reduced hepatocyte turnover), reversal of fibrosis and cirrhosis [58,59], and up to a five-fold reduction in the five year risk of HCC [38], with the greatest reduction occurring in patients with the highest risk factors at study entry [41]. However, in these older patients, the incidence of HCC during therapy still accumulates with time and is not reduced to that of healthy controls, even in those who achieved immune control by age 30, with virus titers below 10^4^ copies/mL and normal ALTs. This raises the possibility that factors predisposing to the later development of HCC may become established in younger patients but only manifest clinically as HCC after many years or even decades, with residual liver turnover and damage promoting the emergence of HCC. Indeed, because immune control typically involves emergence of HBeAg(−) HBV, possibly via immune selection against hepatocytes infected with wtHBV [48], it is likely that some patients in the immune control phase have, by age 30, experienced significant cumulative hepatocyte turnover. This would be even greater in the third of patients with CHB in the REVEAL-HBV study that were HBeAg(−) with virus titers above 10^4^ copies/mL at the time of study entry [27], i.e. that had achieved only partial immune control of replication and still have active hepatitis. A simple interpretation, which raises concerns about leaving IT patients untreated, is that mutations that initiate oncogenic progression may occur early in life. In this situation, even mild enhancement of hepatocyte proliferation provided by immune-mediated killing of infected hepatocytes in IT patients could, over many years, significantly promote progression to HCC. This would be even more severe if young patients enter the immune active phase without detection. Promotion could be even greater if hepatocyte turnover is also selective, facilitating outgrowth of initially rare hepatocytes, including some that are preneoplastic. For instance, the killing of hepatocytes producing wtHBV may be more effective than against those producing HBeAg(−) HBV mutants. Similarly, hepatocytes that carried or acquired mutations that prevented them from supporting HBV replication [8,27,32,60] should not be directly killed by anti-HBV cytotoxic T-lymphocytes (CTLs) and should therefore have a survival advantage, allowing them to expand in number to replace hepatocytes that support HBV replication. This seems likely, as foci and swathes of hepatocytes free of core antigen detectable by immunohistochemistry and replicating HBV as detected by in situ hybridization, emerge during the course of chronic infection [60,61,62], even in the face of ongoing viremia.

Importantly, while patients in the IT phase and younger patients in their first and second decades are often considered synonymous (e.g. [2]), this likely overestimates the incidence of the immune tolerant phase of infection in the young. In one study, approximately one out of three patients had undergone HBeAg seroconversion by age nine, and approximately one of two by age 19 (Figure 2). In addition, fibrosis, reflecting hepatocyte damage, can be detected even in young patients diagnosed as IT, based on HBeAg positivity, high virus titers (≥10^9^ copies/mL), and normal serum ALT [29]. In other words, hepatocyte damage and turnover may be significant in many young carriers, including those believed to be in the IT phase [63]. Moreover, the fact that more than half of chronic infections spontaneously progress to the immune active phase or the immune control phase before the third decade of life [32] implies that a significant amount of transient, or more ominously, persistently elevated hepatocyte turnover may have taken place, even in or before the teenage years. Presumably, the way in which this affects HCC risk depends both upon the duration of active hepatitis and the age at which immune control occurs. The earlier immune control is achieved presumably contributes to a lower long-term risk of HCC.

## 4. Evidence Consistent with Elevated Liver Damage in Immune Tolerant Patients

The concept of an IT phase of HBV infection has been based largely upon persistently normal ALT levels along with high titer viremia of 10^9−10^ copies/mL; that is, since the immune system is not achieving perceptible control of virus replication during this phase [64], the host must be tolerant of the infection. CHB patients, irrespective of disease phase, do indeed present a defective HBV-specific immune response [21,65]. In fact, the quantity of circulating HBV-specific T cells is actually higher in those with resolved acute HBV than in subjects with chronic HBeAg (+) disease. Furthermore, there is no difference in the number of intrahepatic HBV-specific T cells in CHB patients with and without liver inflammation [66].

There is continued debate in the field whether the HBV-specific immune response is contributing to liver damage and disease progression in the IT phase. However, mild liver damage appears common in studies of IT patients. For example, an early, small scale study of liver biopsies and sera from 18 neonatally infected HBsAg(+) children in Taiwan (aged 4–9 years) showed three with chronic persistent hepatitis and 15 with non-specific histologic changes in the liver, i.e. liver abnormalities were found in all 18 children [6]. Likewise, the presence of mild hepatitis and fibrosis in IT patients (e.g. Table 1 of reference [63]), indicative of immune mediated liver damage and the destruction of hepatocytes, underlines the obvious pitfalls of labeling this as a purely benign state of infection. Even though disease is mild, this needs to be understood in terms of the years or decades that patients may remain in the IT phase. In line with these observations, our data, demonstrating that the peripheral HBV-specific T cell response in IT patients was quantitatively and functionally superior to that seen in patients considered to be in the immune active phase of disease [67], challenges the accuracy of clinical categorization of the IT phase as benign.

In addition to immunologic and histologic evidence for active disease in immune tolerant patients, there is also direct and indirect evidence that genetic damage to the hepatocyte population occurs during the IT phase and may be important in oncogenesis. The basis for this claim is two-fold: (1) HBV DNA integration into hepatocyte DNA, at a sufficient frequency to mutate essentially any host gene in at least one hepatocyte in the liver, has been observed [63]; in addition, the frequency of HBV DNA integration probably reflects a much higher level of DNA damage within the hepatocyte population [68]. Thus, mutations that initiate the progression to HCC may occur in the IT phase of infection; (2) since integration into host DNA occurs at random sites, integrated HBV DNA can be used as a cell lineage marker to quantify clonal hepatocyte expansion. Using this approach, the sizes of hepatocyte clones were found to greatly exceed predictions from a model of hepatocyte regeneration based on the current consensus on the nature of normal liver renewal [17,61,62,63], in which hepatocyte death and division are essentially random events in which the hepatocyte population is self-renewing. Some of this clonal expansion must be due to random death and compensatory division that is involved in liver maintenance, even in the healthy liver [69], and some to the random killing by antiviral T cells. However, as discussed below, clones of >1000 hepatocytes in IT patients appear too large to be produced by random non-selective turnover unless very high random turnover rates are presumed. Production of these large clones seems more likely to reflect selective processes [63], such as the emergence of clones of virus resistant hepatocytes and/or of hepatocytes infected with HBeAg(−) HBV (e.g. discussed in reference [48]) that can escape CTL killing. Such processes might promote an HCC initiating mutation that occurred in the IT phase [8].

Other explanations for unexpected amounts of clonal hepatocyte expansion may exist; although all hepatocytes are generally considered to contribute to liver maintenance, it is possible that this property is actually restricted to a subset of hepatocytes. (We assume hepatocytes, since clonal expansion can be detected using integrated HBV DNA as a marker). In this case, cell death might lead to a more rapid genetic narrowing of the hepatocyte population and, accordingly, progression to HCC; for instance, if preneoplastic lesions were introduced into hepatocytes that are uniquely proliferative. As an example of the possibility that only a subset of hepatocytes are proliferative in normal liver maintenance, a recent study provided evidence that hepatocytes surrounding the central vein are the source of hepatocyte renewal in grown mice [70], with daughter cells expanding towards portal areas and replenishing ~40% of the hepatocyte population within a year. While this observation seems at odds with earlier studies in rats and mice, which demonstrated clonal hepatocyte expansion elsewhere in the lobule [71,72], the possibility that this model applies to human liver needs to be considered. This is important because undisputed interpretations of liver turnover and hepatocyte replacement still remain elusive.

## 5. Clonal Hepatocyte Expansion and HCC

As noted above, HBV DNA integration occurs at random sites in host DNA. Thus, proliferation of a hepatocyte lineage can be easily followed by quantifying individual integration sites in a small liver sample [13,17,61,62,68,73,74,75]. Histologic counterparts of some of these hepatocyte clones may be the foci of hepatocytes that emerge during chronic infection that are free of replicating virus, as observed in studies of chimpanzees chronically infected with HBV and woodchucks chronically infected with woodchuck hepatitis virus (WHV) [60]. A possible link between such foci and HCC may be in the observation that preneoplastic foci of altered hepatocytes (i.e. foci of morphologically altered hepatocytes) observed in chronic infections (with HBV and WHV) often appear to be virus negative [76,77,78,79]. That is, the loss of the ability to support HBV replication or HBeAg synthesis [48] may, in theory, give hepatocytes with preneoplastic mutations a survival advantage in progressing to HCC by helping them avoid antiviral CTLs. These hepatocytes clones could arise even more rapidly if mutations that block hepadnavirus replication emerged in a hypothetical subset of hepatocytes that were dedicated to maintenance of liver mass. Indeed, given current and past controversies on hepatocyte replacement in the healthy liver and during chronic liver injury, we need to appreciate that various mechanisms of liver maintenance are still under consideration. It is therefore important to consider, in contrast to the consensus model discussed earlier, the impact of models in which a small subset of hepatocytes is uniquely responsible for maintaining the hepatocyte population. As discussed below, we believe that such models fail to fit experimental observations. Nonetheless, until these issues are completely resolved, interpretation of data on chronic liver injury and renewal will remain inconclusive. Our focus, simply for illustration, is a comparison of the consensus model and the recent evidence from the mouse model that hepatocyte replacement in the absence of acute liver injury is an exclusive function of a niche of differentiated hepatocytes, with stem cell properties, that surround the central vein of the liver lobule [70].

**Figure 3:**
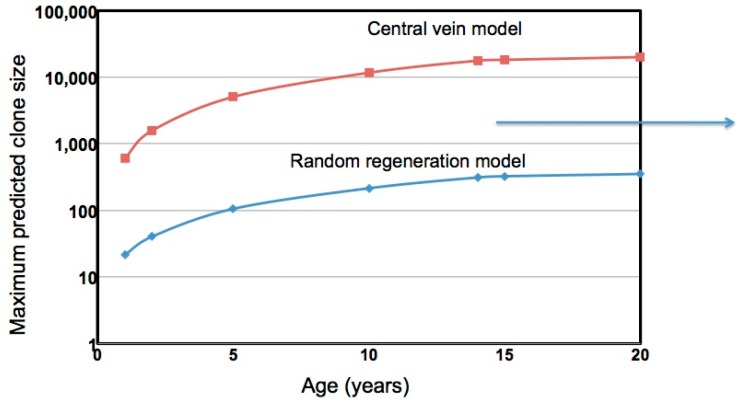
Predictions of hepatocyte clone size based on liver growth and hepatocyte turnover. Two models are compared, one assuming that all hepatocytes contribute to liver growth and hepatocyte turnover (consensus model) [80], the other assuming that proliferation is an exclusive property of hepatocytes surrounding the central vein [70]. In these simulations, hepatocyte clones develop beginning from birth. The liver linearly expands 10-fold in size over a period of 14 years. In both simulations, 0.15% of hepatocytes were replaced daily due to random cell death. Hepatocytes are uniquely marked at time zero to follow their loss and/or clonal expansion. Two FORTRAN computer models of the liver were compared; in the first (csize8), all hepatocytes participate in growth and replacement. In the second (ran-rep-c5), only central vein hepatocytes, considered to represent 1% of total hepatocytes, are able to divide. (Interestingly, if liver growth is not included, then the stem cell model, with 0.15% daily turnover, predicts 42% replacement of the liver with stem cell (central vein hepatocyte) daughters after a year, similar to experimental observations [70]). These FORTRAN models are available upon request from W.S.M. (ws_mason@fccc.edu) or S.L. (S_Litwin@fccc.edu). The right pointing arrow illustrates the average maximum clone size for IT patients ranging from 15 to 39 years of age; no age dependence was observed [63].

A comparison of the predictions of these two models and experimental data are shown in Figure 3. Again, in the consensus model, hepatocyte death and division are random events, division occurring to compensate for cell death to maintain liver size. In the second model, division of the ~1% of hepatocytes surrounding the central vein is the exclusive source of hepatocyte replacement throughout the lobule [70]. All cells, including those at the central vein, are subject to killing at the same rate: 0.15% per day, three times the number of hepatocytes reported to be in the S-phase of the cell cycle in the healthy liver [69]. In the consensus model, the rate of passage into S-phase is considered to reflect the rate of hepatocyte replacement. In the central vein model, this correlation is less clear. Passage into S-phase of hepatocytes other than at the central vein is assumed to be an abortive event that does not lead to new hepatocytes. Nonetheless, the idea that the correlation between S-phase hepatocytes in the lobule and hepatocyte replacement is valid lies in the fact that the assumption of 0.3% replacement per day would lead to expansion of central vein hepatocytes to occupy 40% of the lobule after a year (calculations not shown); for comparison, 42% was found in the mouse model [70].

Figure 3 shows a computation of the maximum hepatocyte clone sizes expected over time up to 20 years, assuming liver size expands 10-fold in the first 14 years of life. For comparison, we have noted (arrow) an average maximum clone size of 1200–1300 hepatocytes that we previously detected in a study of integrated HBV DNA in hepatocytes of IT patients [63]. As shown, using the assumption that new hepatocytes are produced entirely from a central vein compartment encompassing 1% of hepatocytes, maximum clone sizes of 10,000 or more hepatocytes are expected, much larger than those actually observed. This discrepancy appears to fit with other data, such as S-phase measurements, which suggest that the central vein stem cell model may not apply to human liver. In contrast, the consensus model, involving random death and regeneration, underestimates the maximum clone sizes that are observed, unless we assume much higher rates of liver turnover [63] or perhaps a selective process involving, for instance, immune escape.

How the stem cell model can be aligned with evidence suggesting that hepatocytes throughout the hepatic lobule are able to proliferate in the healthy adult liver remains unclear. As noted, hepatocytes throughout the lobule can be found in S-phase during chronic hepatitis B, not just those around the central vein. The idea is also at odds with a study in rats showing the focal expansion of non-central vein hepatocytes that have been marked via infection by a retrovirus vector expressing a beta-galactosidase marker gene [71] and with a study of tagged hepatocytes in mice [72]. Conversely, the evidence for hepatocyte replacement originating from the hepatocytes surrounding the central vein seems very convincing for normal mouse liver when taken by itself [70], and this issue should be a major focus of future research. In brief, whether or not this is a mouse specific phenomenon needs to be determined, especially with regard to the overall contribution of this compartment to hepatocyte renewal in human liver, since any interpretation of disease progression during chronic hepatitis B is crucially linked to the proliferative potential of hepatocytes in the infected liver.

Indeed, other studies show that human hepatocytes transplanted to mice can undergo extensive proliferation, including those with markers of senescence [80,81,82,83,84]. Importantly, such studies also appear to rule out stem cells in the canals of Hering as a major contributor to hepatocyte replacement, even in cases of extensive liver damage, and suggest instead that proliferating hepatocyte progenitors, which are detected in this situation, are formed by the dedifferentiation of mature hepatocytes [82,85].

Based upon available evidence, we favor the current consensus that the vast majority of hepatocytes have the ability to divide to maintain the hepatocyte population during chronic liver disease. The potential for clonal hepatocyte expansion in this model to contribute to the development of HCC lies in the fact that the hepatocyte population is self-renewing and therefore closed. Thus, mutant hepatocytes that underwent even random clonal expansion would become harder to totally eliminate over time as their clone size increased. Although we don’t favor the central vein model for reasons discussed above, we accept that the same argument for the importance of a closed population in disease progression could be applied to central vein hepatocytes.

In brief, in both models, normal or enhanced hepatocyte turnover (e.g. via CTL killing), will lead to the loss of some hepatocyte lineages and the expansion of others over time. In addition, the process of clonal expansion would be greatly enhanced if some hepatocytes escape immune killing (mediated by anti-HBV T cells or antibodies), as proposed for the emergence of HBeAg(−) variants of HBV [48] (i.e. due to differential immune selection against hepatocytes producing wtHBV). An important example of clonal hepatocyte expansion via immune selection may lie in the observation that the fraction of productively infected human hepatocytes declines over time during chronic HBV infection, based on staining for viral core antigen and/or in situ hybridization for replicative viral DNA. This can occur even in the face of a high titer viremia ([86] for review). Histologic data suggest that this process affects hepatocyte lineages (clones) not just isolated hepatocytes, as virus free hepatocytes can emerge in a focal distribution suggestive of clonal expansion (e.g. as observed in the chimpanzee model of HBV infection [60]). Again, histologic evidence appears to favor the consensus model, especially outside the mouse. 

In closing, while the focus here is on chronic HB, we note that clonal hepatocyte expansion, aka clonal hepatocyte repopulation, is considered a major HCC risk factor in genetic diseases of the liver [87].

## 6. HBV Vaccine and Prospects for HBV Elimination

Although our main focus has been on the time course of progression of chronic hepatitis B and, by inference, more aggressive clinical monitoring focused on the possible need for earlier treatment, it is important to note that the high cost of treatment limits widespread application. Thus, there is a major need for a cheap and effective cure for chronic HBV infection. Whether the research community has the critical personnel and material resources to achieve an HBV cure in the next 5–10 years is debatable.

Indeed, emphasis on HBV as an important public health problem has been mitigated by the perception that the HBV vaccine, available since the late 1970s [88,89], could lead to the elimination of incident cases of HBV globally. For instance, the World Health Organisation (WHO) has estimated that HBV could be eliminated as a major public health problem by 2030. The strategy outlines ambitious aims to reduce the incidence of chronic hepatitis B cases globally from the current estimates of 6–10 million to 0.9 million by 2030 and consequently reduce the deaths attributable to HBV (primarily due to HCC) from 1.4 million to 0.5 million [90].

WHO recommends universal immunisation against HBV in infancy [91], and, by the end of 2015, 185 countries had adopted this strategy, resulting in 83% of infants being successfully immunised globally in an intensive effort to reduce the global burden of CHB [92]. There is no doubt that immunisation programmes can have a meaningful impact on patient outcomes. Robust data collection from Taiwan has demonstrated the efficacy of adoption of a universal vaccination program, with 85% of infants born to highly infectious mothers producing adequate levels of anti-HBs following a dose of hepatitis B immunoglobulin (HBIG) and vaccination against hepatitis B commencing within 24 h of birth [93]. The efficacy of mass vaccination programmes has been confirmed in other settings such as in The Gambia, where the prevalence of chronic hepatitis B was reduced from 12.4% to 0.8% [94,95,96]. In Tunisia, the universal immunisation programme has resulted in 68.9% of those immunised carrying the protection of levels of anti-HBs >10 mIU/mL into early adulthood [97], whereas a vaccine programme at 6, 10, and 14 weeks has resulted in sero-protection in 87% of immunised infants in South Africa [98]. Data from Taiwan importantly demonstrates the efficacy of a universal immunisation programme in reducing the longer-term risk of HCC [99].

Universal immunisation protects from horizontal transmission in infancy, but, to protect from perinatal mother to child transmission (MTCT), a birth dose vaccine is required within 24 h of delivery. This is particularly relevant in endemic areas where HBeAg positivity can persist into childbearing years, rendering the mother highly infectious. HBeAg loss at an earlier age in Sub-Saharan African populations makes MTCT only accountable for 10% of CHB [100,101] compared with an estimated 40% of total infections in China [102]. Those women in Africa who are highly infectious are often those co-infected with HIV, in whom the risk of MTCT is increased further [100,103]. At present, only 96 countries include a birth dose vaccine in the immunisation programme, equating to the protection of around 39% of new born babies [92]. Some low prevalence nations such as the UK have not adopted universal immunisation against HBV and offer instead antenatal screening for blood borne viruses and immunisation to infants born to HBV-infected mothers. The birth dose vaccine was administered to 100% of babies born to HBsAg positive mothers in this developed healthcare system [104], but completion of immunisation occurred in only 83% of cases [104,105]. Even if HBIG and vaccination are given as birth doses, there is an estimated 8–30% risk of MTCT in highly infectious mothers [106]. Failure of immunoprophylaxis was observed in around 5% of children born to HBeAg positive mothers in a Chinese population. Inadequate initial injections and HBV DNA ≥ 5 × 10^8^/mL were independently associated with an increased risk of MTCT [107]. Around 20–30% of infected children in Taiwan, who received appropriate immunoprophylaxis, were found to have HBsAg mutant virus [108]. Failure of immunoprophylaxis has implications at the population level in view of the selection pressure to favour HBV variants with surface antigen mutations [109,110], although it is not thought that mutant variants will become dominant over wild type virus following vaccination for at least half a century [111].

The use of Tenofovir in the third trimester has been shown to significantly reduce this risk of MTCT from highly viraemic women [112]. Data pertaining to the public health impact of the earlier initiation of treatment in patients in the IT phase of disease through the reduction of MTCT is lacking. In practice, more widespread treatment strategies may be difficult to deliver in areas of endemicity, where one would assume the impact to be greatest.

In resource-limited settings, the delivery of effective immunisation programs is often challenging and limited by a lack of funding and poor infrastructure for healthcare delivery in remote communities [113]. Conflict zones face specific challenges [114,115]. The vaccination program in China was co-funded by GAVI, the global Vaccine Alliance, and is an example of a successful partnership programme resulting in excellent vaccination coverage [102]. A lack of public knowledge and ongoing stigma surrounding HBV will pose barriers in some communities, regardless of national wealth [63,116,117]. Many of these challenges are not applicable in developed Western nations, yet effective delivery of both targeted and universal vaccination programmes still appears to remain suboptimal [104,118,119]. Further large-scale investment will be needed globally to eliminate HBV as a major public health problem [120].

The aspiration of the WHO to eliminate CHB as a global health problem by 2030 is noble but not without significant challenges. Whether it is feasible to translate this aspiration into a reality remains to be seen. Despite the availability of a highly effective prophylactic vaccine, the problems encountered with vaccine delivery re-emphasize the need for intense research efforts leading to an HBV cure.

## 7. Summary and Conclusions

The ultimate goal of HBV research and treatment is to produce a rapidly acting cure for chronic infections. Ideally, the cure should work in children, to minimize the risk of liver lesions that can progress to HCC. In the shorter term, the goal is to use existing therapies to achieve the same goal, to prevent the development of cirrhosis and HCC by using therapies that that block HBV replication, but in the vast majority of cases these do not lead to a cure.

We have attempted to document herein reasons to consider evaluating current approaches to CHB, including the provision of nucleos(t)ide analog therapy to young patients irrespective of serologic status and including those characterized as IT. In contrast, current guidelines generally see cirrhosis and HCC as diseases for which prevention via antiviral therapy should only be considered after there is clear evidence of active hepatitis, particularly elevated ALTs, for a period of time or evidence of established liver damage on biopsy. From our perspective, this has at least three major limitations: (1) the need to maintain active clinical surveillance over many years to determine when there is a persistent rise in ALTs, justifying therapy; (2) the need, not readily met in clinical practice, to also perform regular liver biopsies of patients with normal ALTs to look for liver damage not detectable by current serology; (3) the fact that the current clinical approach reduces the rate of but does not eliminate the progression to HCC, even though progression to cirrhosis is blocked; and (4) the low therapeutic coverage (<1% worldwide) already implicit in the current approach to CHB management that requires constant clinical monitoring and does little more than slow the progression to HCC in those selected for treatment. Young people with CHB will continue to present a major global healthcare challenge until universal vaccination is properly administered and completely effective.
